# Supplementary material for: Complex Tissue Regeneration in Mammals Is Associated With Reduced Inflammatory Cytokines and an Influx of T Cells
Source: Front Immunol. 2020 Aug 7;11:1695. doi: 10.3389/fimmu.2020.01695 (PMC7427103; doi:10.3389/fimmu.2020.01695)
Supplement: Supplementary file 1 [file Data_Sheet_1.PDF]

## SUPPLEMENTAL MATERIALS

Figure S1. Comparison of parallelism for cytokine assays demonstrates ability to quantify cytokines across three species.

Figure S2. The tissue microenvironment is dynamic, and the ability to regenerate is associated with a specific immune response to injury.

Table S1. Summary of animals used in this study.

Table S2. One-way ANOVA analyses of log-transformed uninjured serum data.

Table S3. Two-way ANOVA analyses of log-transformed serum time series data.

Table S4. Two-way ANOVA analyses of log-transformed tissue lysate time series data.

Table S5. One-way ANOVA analysis of tissue cytokine data to test for an effect of group at D1, 2, 10, 20.

Table S6. List of T cell phenotyping antibodies tried using flow cytometry in *Acomys cahirinus*.

Data File S1 contains summary tables of post-hoc multiple comparison tests used in Figure 2, Figure 3 and Figure S2.

Data File S2**.** Comparisons of amino acid sequence alignments for antigens in study.

## SUPPLEMENTAL FIGURES

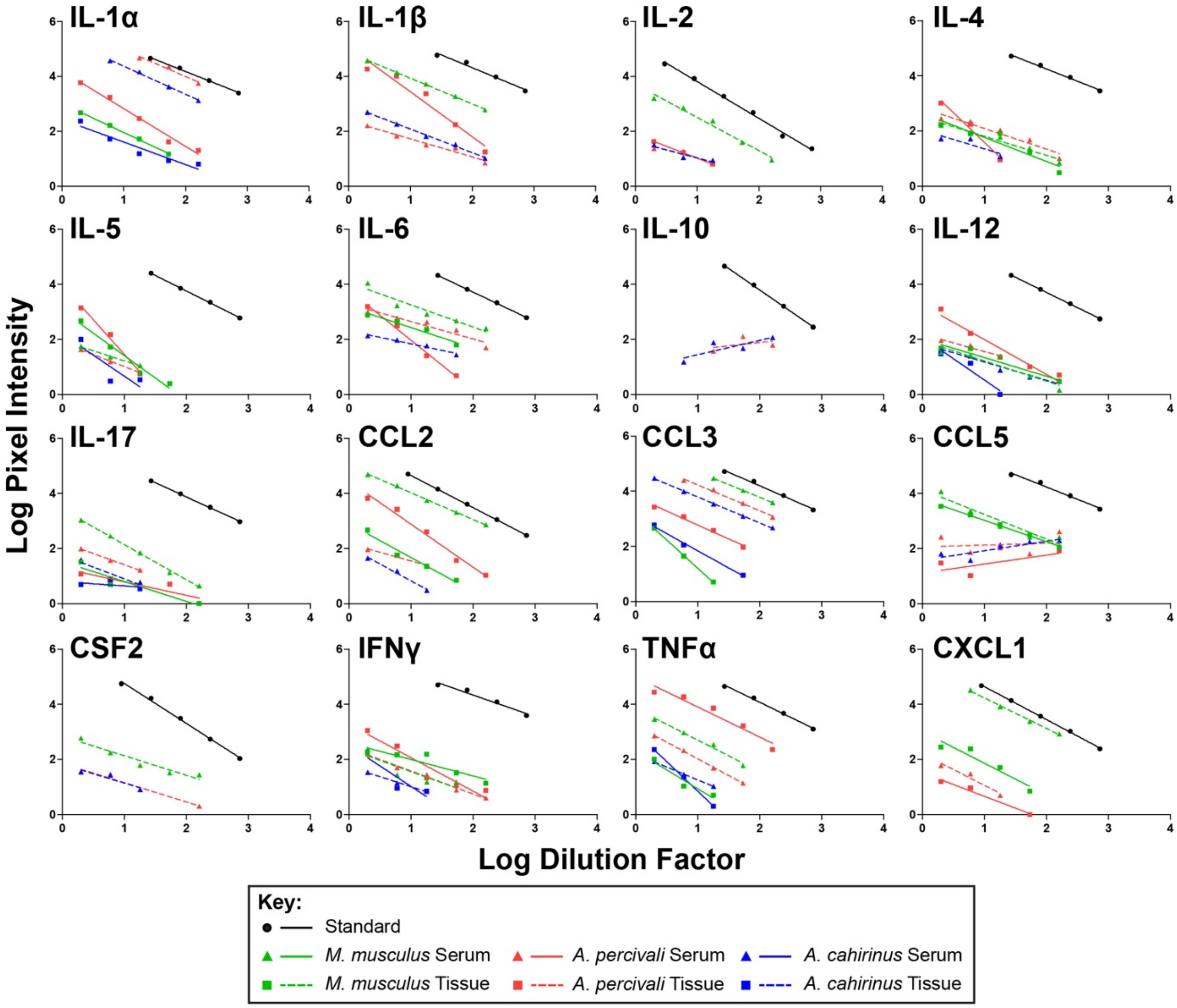


**Figure S1. Comparison of parallelism for cytokine assays demonstrates ability to quantify cytokines across three species.** Serial dilution of serum (triangles, solid lines) and tissue lysate (squares, dashed lines) shows similar negative slopes for *M. musculus* (green), *A. percivali* (red) and *A. cahirinus* (blue) compared to the standard (black, circles, solid line). Data represent the mean for duplicates and lines are linear regressions calculated from all data that was above the lower limit of detection.

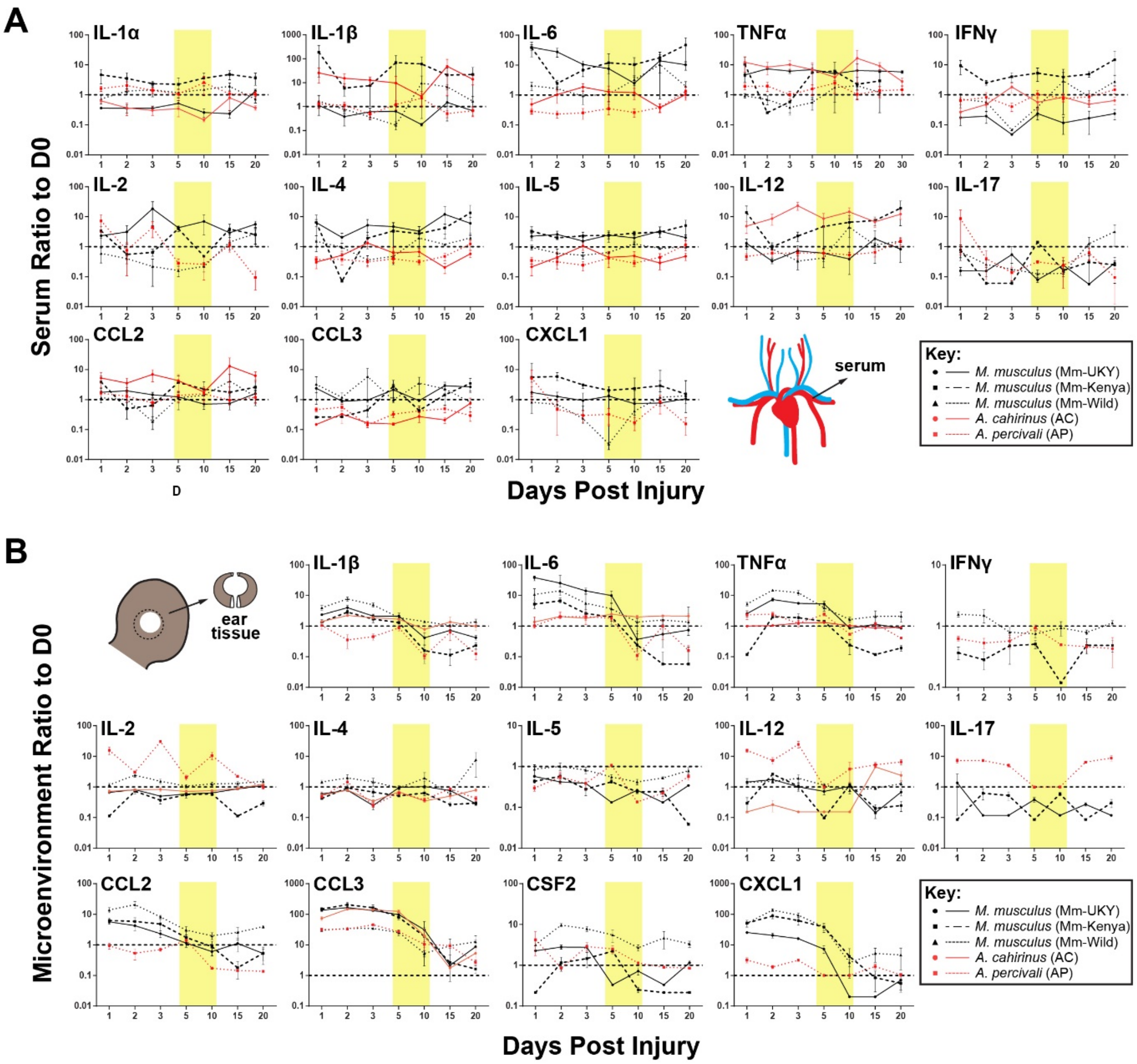


**Figure S2. The tissue microenvironment is dynamic, and the ability to regenerate is associated with a specific immune response to injury. (A)** The change in systemic cytokines compared to D0 over 20 days after ear hole punch. A naïve immune system response (solid lines) was consistent with increased IL-2 and TNFα, and decreased IL-1α compared to a primed response (intermittent lines) (See Table S3). Fibrotic repair (black) was associated with increases in IL-5, IL-6, IL-17, CCL3 and CXCL1 compared to regeneration (red). **(B)** Comparative cytokine changes from tissue lysate compared to D0 over 20 days after ear hole punch. All cytokines varied over time and between groups (See Table S4). There was a stronger increase in CCL3 and a muted increase for IL-12 and CXCL1 in immune-naïve (solid lines) compared to immune-primed animals (intermittent lines). Non-regenerators (black) had stronger increases for IL-6, CCL2 and CXCL1 compared to regenerators (red). Additionally, IL-17 decreased in non-regenerators and increased in regenerators. Data represent mean and S.E.M. for at least n=5 animals per species per timepoint. The dashed line at Y=1 represents no change compared to D0 and the yellow boxes represent the inflammation resolution window.

## SUPPLEMENTAL TABLES

**Table S1**: Summary of animals used in this study.

| | *M. musculus* | *M. musculus* | *M. musculus* | *A. cahirinus* | *A. percivali* |
|---|---|---|---|---|---|
| Abbreviation: | Mm-UKY | Mm-Kenya | Mm-Wild | Ac | Ap |
| Strain: | ND4 | "Swiss" | Wild-caught | UKY Colony | Wild-caught |
| Location: | Kentucky | Kenya | Kentucky | Kentucky | Kenya |
| Life history: | Lab-reared | Local breeder | Wild | Lab-reared | Wild |
| Regenerates? | No | No | No | Yes | Yes |
| Immune status: | Naïve | Primed | Primed | Naïve | Primed |
| Animal numbers by experiment in this study: | | | | | |
| **Parallelism** (In addition to pooled samples) | 3 (Female)<br>3 (Male) | n/a | n/a | 3 (Female)<br>3 (Male) | 3 (Female)<br>3 (Male) |
| **Cytokine analysis time course** | | | | | |
| Per timepoint: | 3 to 5 (F)<br>3 to 5 (M) | 5 to 9 (F) | 1 to 3 (F)<br>2 to 4 (M) | 3 to 5 (F)<br>3 to 5 (M) | 5 to 7 (F)<br>3 to 5 (M) |
| Totals: | 28 (F)<br>30 (M) | 59 (F) | 16 (F)<br>23 (M) | 30 (F)<br>30 (M) | 41 (F)<br>28 (M) |
| **T cell analysis** | | | | | |
| Flow: | 22 (F) | n/a | n/a | 21 (F) | n/a |
| Histo. / Western: | 28 (F) | n/a | n/a | 28 (F) | n/a |
| **COX-2 inhibition** | | | | | |
| D10 Histology: | 10 (F) | n/a | n/a | n/a | n/a |
| Time course: | 20 (F) | n/a | n/a | n/a | n/a |

**Table S2**: One-way ANOVA analyses of log-transformed uninjured serum data. Groups listed in comparison column represent groups for which data was quantified and could be compared. *P*-values indicate where at least one group is significantly different from another group. Tukey-Kramer HSD post-hoc tests were used for pairwise comparisons are summarized in Figure 1A (See Data File S1).

| **Antigen** | **Comparison** | **N** | **F** | ***P*-value** |
|---|---|---|---|---|
| IL-1α | All groups | 5 | 2.69 | 0.051 |
| IL-1β | All groups | 5 | 11.15 | <0.001* |
| IL-2 | All groups | 5 | 10.27 | <0.001* |
| IL-4 | All groups | 5 | 6.00 | 0.001* |
| IL-5 | All groups | 5 | 2.63 | 0.055 |
| IL-6 | All groups | 5 | 6.31 | 0.001* |
| IL-12 | All groups | 5 | 5.21 | 0.003* |
| IL-17 | Mm-UKY, Mm-Kenya, Mm-Wild, Ap | 4 | 1.15 | 0.349 |
| CSF2 | None – all undetectable | n/a | n/a | n/a |
| CCL2 | All groups | 5 | 16.03 | <0.001* |
| CCL3 | All groups | 5 | 2.59 | 0.058 |
| IFNγ | All groups | 5 | 3.25 | 0.026* |
| TNFα | All groups | 5 | 23.80 | <0.001* |
| CXCL1 | All groups | 5 | 4.07 | 0.010* |

**Table S3**: Two-way ANOVA analyses of log-transformed serum time series data. Groups listed in comparison column represent groups for which data was quantified throughout the time series. Tukey-Kramer HSD post-hoc tests were used for pairwise comparisons and the results are summarized in Figure 1B (See Data File S1).

| | | | **Group** | | **Day** | | **Group*Day** | |
|---|---|---|---|---|---|---|---|---|
| **Antigen** | **Comparison** | **N** | **F** | ***P*-value** | **F** | ***P*-value** | **F** | ***P*-value** |
| IL-1α | All groups | 255 | 32.51 | <0.001* | 0.93 | 0.471 | 1.62 | 0.039* |
| IL-1β | Mm-UKY, Mm-Wild, Ap | 157 | 3.02 | 0.052 | 1.06 | 0.391 | 1.08 | 0.386 |
| IL-2 | All groups | 255 | 27.82 | <0.001* | 2.46 | 0.025* | 1.96 | 0.006* |
| IL-4 | All groups | 255 | 34.54 | <0.001* | 1.47 | 0.188 | 1.55 | 0.053 |
| IL-5 | All groups | 255 | 63.22 | <0.001* | 0.94 | 0.469 | 0.82 | 0.706 |
| IL-6 | All groups | 255 | 64.61 | <0.001* | 2.29 | 0.037* | 2.63 | <0.001* |
| IL-12 | All groups | 255 | 33.25 | <0.001* | 2.21 | 0.660 | 0.91 | 0.583 |
| IL-17 | Mm-UKY, Mm-Kenya, Mm-Wild, Ap | 205 | 7.64 | <0.001* | 2.78 | 0.013* | 2.60 | <0.001* |
| CSF2 | None – all undetectable | 0 | n/a | n/a | n/a | n/a | n/a | n/a |
| CCL2 | All groups | 255 | 5.72 | <0.001* | 1.46 | 0.193 | 1.16 | 0.278 |
| CCL3 | All groups | 255 | 38.75 | <0.001* | 0.91 | 0.486 | 1.47 | 0.079 |
| IFNγ | All groups | 255 | 37.81 | <0.001* | 1.22 | 0.296 | 1.77 | 0.018* |
| TNFα | All groups | 255 | 31.88 | <0.001* | 0.85 | 0.535 | 0.91 | 0.583 |
| CXCL1 | Mm-UKY, Mm-Kenya, Mm-Wild, Ap | 205 | 69.43 | <0.001* | 4.33 | <0.001* | 1.47 | 0.104 |

**Table S4**: Two-way ANOVA analyses of log-transformed tissue lysate time series data. Groups listed in comparison column represent groups for which data was quantified throughout the time series. *P*-values indicate where at least one group is significantly different from another group. Tukey-Kramer HSD post-hoc tests were used for pairwise comparisons and the results are summarized in Figure 2 (See Data File S1).

| | | | **Group** | | **Day** | | **Group*Day** | |
|---|---|---|---|---|---|---|---|---|
| **Antigen** | **Comparison** | **N** | **F** | ***P*-value** | **F** | ***P*-value** | **F** | ***P*-value** |
| IL-1α | None – all above limit of detection | 0 | n/a | n/a | n/a | n/a | n/a | n/a |
| IL-1β | All groups | 261 | 77.88 | <0.001* | 39.47 | <0.001* | 5.33 | <0.001* |
| IL-2 | All groups | 261 | 190.93 | <0.001* | 6.68 | <0.001* | 13.59 | <0.001* |
| IL-4 | All groups | 261 | 29.09 | <0.001* | 11.24 | <0.001* | 6.76 | <0.001* |
| IL-5 | Mm-UKY, Mm-Kenya, Mm-Wild, Ap | 211 | 22.73 | <0.001* | 10.03 | <0.001* | 10.70 | <0.001* |
| IL-6 | All groups | 261 | 76.89 | <0.001* | 104.11 | <0.001* | 15.06 | <0.001* |
| IL-12 | All groups | 261 | 123.20 | <0.001* | 10.42 | <0.001* | 18.28 | <0.001* |
| IL-17 | Mm-UKY, Mm-Kenya, Ap | 177 | 598.63 | <0.001* | 4.42 | <0.001* | 17.55 | <0.001* |
| CSF2 | Mm-UKY, Mm-Kenya, Mm-Wild, Ap | 211 | 104.44 | <0.001* | 21.36 | <0.001* | 8.51 | <0.001* |
| CCL2 | Mm-UKY, Mm-Kenya, Mm-Wild, Ap | 211 | 72.96 | <0.001* | 38.71 | <0.001* | 5.23 | <0.001* |
| CCL3 | All groups | 261 | 8.30 | <0.001* | 160.11 | <0.001* | 6.24 | <0.001* |
| IFNγ | Mm-Kenya, Mm-Wild, Ap | 161 | 40.47 | <0.001* | 2.42 | 0.030* | 3.00 | <0.001* |
| TNFα | All groups | 261 | 149.87 | <0.001* | 81.32 | <0.001* | 11.84 | <0.001* |
| CXCL1 | Mm-UKY, Mm-Kenya, Mm-Wild, Ap | 211 | 137.76 | <0.001* | 201.75 | <0.001* | 26.64 | <0.001* |

**Table S5**: One-way ANOVA analysis of tissue cytokine data to test for an effect of group at D1, 2, 10 and 20. *P*-values indicate where at least one group is significantly different from another group. Tukey-Kramer HSD post-hoc tests were used for pairwise comparisons and are summarized in Figure 3 (See Data File S1).

| **Antigen** | **Comparison** | **N** | **Day** | **F-Ratio** | ***P*-value** |
|---|---|---|---|---|---|
| IL-1β | All groups | | 1 | 5.39 | 0.002* |
| | | | 2 | 32.52 | <0.001* |
| | | | 10 | 20.70 | <0.001* |
| | | | 20 | 19.05 | <0.001* |
| IL-4 | All groups | | 1 | 9.71 | <0.001* |
| | | | 2 | 4.8 | 0.004* |
| | | | 10 | 5.23 | 0.003* |
| | | | 20 | 18.89 | <0.001* |
| IL-6 | All groups | | 1 | 14.56 | <0.001* |
| | | | 2 | 47.24 | <0.001* |
| | | | 10 | 23.30 | <0.001* |
| | | | 20 | 21.50 | <0.001* |
| IL-12 | All groups | | 1 | 57.33 | <0.001* |
| | | | 2 | 14.22 | <0.001* |
| | | | 10 | 7.66 | <0.001* |
| | | | 20 | 26.33 | <0.001* |
| IL-17 | Mm-UKY, Mm-Kenya and Ap | | 1 | 45.87 | <0.001* |
| | | | 2 | 93.60 | <0.001* |
| | | | 10 | 441.78 | <0.001* |
| | | | 20 | 116.93 | <0.001* |
| CCL2 | Mm-UKY, Mm-Kenya, Mm-Wild, Ap | | 1 | 21.55 | <0.001* |
| | | | 2 | 33.43 | <0.001* |
| | | | 10 | 7.04 | 0.002* |
| | | | 20 | 11.68 | <0.001* |
| TNFα | All groups | | 1 | 39.61 | <0.001* |
| | | | 2 | 54.97 | <0.001* |
| | | | 10 | 27.95 | <0.001* |
| | | | 20 | 67.67 | <0.001* |
| CXCL1 | All groups | | 1 | 60.99 | <0.001* |
| | | | 2 | 210.02 | <0.001* |
| | | | 10 | 15.23 | <0.001* |
| | | | 20 | 9.54 | <0.001* |

**Table S6**: List of T cell phenotyping antibodies tried using flow cytometry in *Acomys cahirinus*.

| Antigen | Clone | Supplier | Catalog# | Status |
|---|---|---|---|---|
| CD45 | OX-1 | BioLegend | 202207 | No – no separation |
| | 30-F11 | BioLegend | 103106 | No – no separation |
| | I3/2.3 | Molecular Probes | A15395 | No – no separation |
| CD3 | 17A2 | BioLegend | 100236 | Yes – good population |
| TCRab | H57-597 | BioLegend | 109207 | No – poor separation |
| | R73 | BioLegend | 201107 | No – no separation |
| TCRgd | GL3 | BioLegend | 118107 | No – poor separation |
| | VC7-13D5 | BioLegend | 107507 | No – poor separation |
| CD4 | GK1.5 | BioLegend | 100433 | No – no separation |
| | RM4-4 | BioLegend | 116011 | No – poor separation |
| CD8 | 53-6.7 | BioLegend | 100726 | No – no separation |
| CD25 | P61 | BioLegend | 102015 | No – poor separation |
| | 3C7 | BioLegend | 101915 | No – no separation |
| CD69 | H1.2F3 | BioLegend | 104511 | No – poor separation |
| CD196 | 29-2L17 | BioLegend | 129813 | No – poor separation |
| | 140706 | BD Pharmingen | 561753 | Yes – good population |
| CD206 | C068C2 | BioLegend | 141705 | Yes – good population |
| CD49b | HMa2 | BioLegend | 103515 | Yes – good population |
| | DX5 | BioLegend | 108909 | No – poor separation |

**Supplemental Files**

Data File S1 contains summary tables of post-hoc multiple comparison tests used in Figure 2, Figure 3 and Figure S2.

Data File S2**.** Comparisons of amino acid sequence alignments for antigens in study. Predicted amino acid sequence was translated from mRNA (*A. cahirinus*) or obtained from NCBI (*H. sapiens*, *M. musculus*, *R. rattus*) and aligned together using MAFFT. Figures were created using BOXSHADE where solid black shading is equivalent, gray shading is similar and no shading is dissimilar.
