# Supplementary material for: Complex Tissue Regeneration in Mammals Is Associated With Reduced Inflammatory Cytokines and an Influx of T Cells
Source: Front Immunol. 2020 Aug 7;11:1695. doi: 10.3389/fimmu.2020.01695 (PMC7427103; doi:10.3389/fimmu.2020.01695)
Supplement: Supplementary file 3 [file Table_2.DOCX]

Supplemental File 2

**IL-1α**

MAFFT Alignment

Homo 1 MAKVPDMFEDLKNCYSENEEDSSSIDHLSLNQKSFYHVSYGPLHEGCMDQSVSLSISETS
Mus 1 MAKVPDLFEDLKNCYSENEDYSSAIDHLSLNQKSFYDASYGSLHETCTDQFVSLRTSETS
Rattus 1 MAKVPDLFEDLKNCYSENEEYSSAIDHLSLNQKSFYDASYGSLHENCTDKFVSLRTSETS
Acomys 1 MAKVPDLFEDLKNCYSENEEYSSAIDHLSLNQKSFYDASYGSGPEHCTAKLVSRRASETS


Homo 61 KTSKLTFKESMVVV---ATNGKVLKKRRLSLSQSITDDDLEAIANDSEEEIIKPRSAPFS
Mus 61 KMSNFTFKESRVTVSATSSNGKILKKRRLSFSETFTEDDLQSITHD-LEETIQPRSAPYT
Rattus 61 KMSTFTFKESRVVVSATSNKGKILKKRRLSFNQPFTEDDLEAIAHD-LEETIQPRSAPHS
Acomys 61 KKSNFTFKESLVMVSATANEGKVLKKRRLSFNQAFDEDDLEAIAHN-LEETIQPRSAPYS


Homo 118 FLSNVKYNFMRIIKYEFILNDALNQSIIRANDQ-YLTAAALHNLDEAVKFDMGAYKSSKD
Mus 120 YQSDLRYKLMKLVRQKFVMNDSLNQTIYQDVDKHYLSTTWLNDLQQEVKFDMYAYSSGGD
Rattus 120 FQNNLRYKLIRIVKQEFIMNDSLNQNIYVDMDRIHLKAASLNDLQLEVKFDMYAYSSGGD
Acomys 120 FQHNVRYKLLRIIKQEFILNDPLNQNIYLDPDNVHLKAASLTDLQHEVKFDMYAYSS-GD


Homo 177 DAKITVILRISKTQLYVTAQDEDQPVLLKEMPEIPKTITGSETNLLFFWETHGTKNYFTS
Mus 180 DSKYPVTLKISDSQLFVSAQGEDQPVLLKELPETPKLITGSETDLIFFWKSINSKNYFTS
Rattus 180 DSKYPVTLKVSNTQLFVSAQGEDKPVLLKEIPETPKLITGSETDLIFFWEKINSKNYFTS
Acomys 179 DSKYPVTLKISNTQLFVSAQGEDQPVLLKEMPEIPKLITGSETNLIFFWKSINSKNYFTS


Homo 237 VAHPNLFIATKQDYWVCLAGGPPSITDFQILENQA
Mus 240 AAYPELFIATKEQSRVHLARGLPSMTDFQI----S
Rattus 240 AAFPELLIATKEQSQVHLARGLPSMIDFQI----S
Acomys 239 AAYPELFIATKEQSRVHLARGLPSMTDFQI----A

**IL-1β**

MAFFT Alignment

Homo 1 MAEVPELASEMMAYYSGNEDDLFFEADGPKQMKCSFQDLDLCPLDGGIQLRISDHHYSKG
Mus 1 MATVPELNCEMPPFDS-DENDLFFEVDGPQKMKGCFQTFDLGCPDESIQLQISQQHINKS
Rattus 1 MATVPELNCEIAAFDS-EENDLFFEADRPQKIKDCFQALDLGCPDESIQLQISQQHLDKS
Acomys 1 MATVPELNSEVTAFHS-DKNDLFFEVDRPQKMKSCLQTLDLGSPDESIQLQISQQHFNKS


Homo 61 FRQAASVVVAMDKLRKMLVPCPQTFQENDLSTFFPFIFEEEPIFFDTWDNEA--YVHDAP
Mus 60 FRQAVSLIVAVEKLWQLPVSFPWTFQDEDMSTFFSFIFEEEPILCDSWDDDDNLLVCDVP
Rattus 60 FRKAVSLIVAVEKLWQLPMSCPWSFQDEDPSTFFSFIFEEEPVLCDSWDDDD-LLVCDVP
Acomys 60 FRQVVSLFVAVEKLWNIPVACPWTFQDEDLGTFFSFIFEEEHILSDSWDDEQ--LVCDVA


Homo 119 VRSLNCTLRDSQQKSLVMSGPYELKALHLQGQDMEQQVVFSMSFVQGEESNDKIPVALGL
Mus 120 IRQLHYRLRDEQQKSLVLSDPYELKALHLNGQNINQQVIFSMSFVQGEPSNDKIPVALGL
Rattus 119 IRQLHCRLRDEQQKCLVLSDPCELKALHLNGQNISQQVVFSMSFVQGETSNDKIPVALGL
Acomys 118 IRQLHCRLRDEQQKCLVLSDPCELKALHLNGENINQQVVFSMSFVHGETSINKIPVALGL


Homo 179 KEKNLYLSCVLKDDKPTLQLESVDPKNYPKKKMEKRFVFNKIEINNKLEFESAQFPNWYI
Mus 180 KGKNLYLSCVMKDGTPTLQLESVDPKQYPKKKMEKRFVFNKIEVKSKVEFESAEFPNWYI
Rattus 179 KGKNLYLSCVMKDGTPTLQLESVDPKQYPKKKMEKRFVFNKIEVKTKVEFESAQFPNWYI
Acomys 178 KGKNLYLSCVMKDGKPTLQLESVDPKQYPKKKMEKRFVFNKTEIKSKVEFESAQFPNWYI


Homo 239 STSQAENMPVFLGGTKGGQDITDFTMQFVSS
Mus 240 STSQAEHKPVFL-GNNSGQDIIDFTMESVSS
Rattus 239 STSQAEHRPVFL-GNSNGRDIVDFTMEPVSS
Acomys 238 STSQAEHKPVFL-GNNSGQDIVDFTMESVSS

**IL-2**

MAFFT Alignment

Homo 1 MYRMQLLSCIALSLALVTNSAPTSSST--------------KKTQLQLEHLLLDLQMILN
Mus 1 MYSMQLASCVTLTLVLLVNSAPTSSSTSSSTAEAQQQQQQQQQQQQHLEQLLMDLQELLS
Rattus 1 MYSMQLASCVALTLVLLVNSAPTSSPA--------------KETQQHLEQLLLDLQVLLR
Acomys 1 MYSMQLASCVALTLVLLVNSAPTSSST--------------EETGQHLEQLLLDLQVLER


Homo 47 GINNYKNPKLTRMLTFKFYMPKKATELKHLQCLEEELKPLEEVLNLAQSKNFHLR-PRDL
Mus 61 RMENYRNLKLPRMLTFKFYLPKQATELKDLQCLEDELGPLRHVLDLTQSKSFQLEDAENF
Rattus 47 GIDNYKNLKLPMMLTFKFYLPKQATELKHLQCLENELGALQRVLDLTQSKSFHLEDAGNF
Acomys 47 GINNYKNPKLPMMLRFKFYMPTKATELKHLQCLEEELGALQSVLDLNQSKSFYLEDTGNF


Homo 106 ISNINVIVLELKGSETTFMCEYADETATIVEFLNRWITFCQSIISTLT-
Mus 121 ISNIRVTVVKLKGSDNTFECQFDDESATVVDFLRRWIAFCQSIISTSPQ
Rattus 107 ISNIRVTVVKLKGSENKFECQFDDEPATVVEFLRRWIAICQSIISTMTQ
Acomys 107 INNIRVTVVKLKGSENTFKCKFDDEPVTVVELLSRWIAFCQSAISTMIQ

**IL-4**

MAFFT Alignment

Homo 1 MGLTSQLLPPLFFLLACAGNFVHGHKCDITLQEIIKTLNSLTEQKTLCTELTVTDIFAAS
Mus 1 MGLNPQLVVILLFFLECTRSHIHGCD-KNHLREIIGILNEVTGEGTPCTEMDVPNVLTAT
Rattus 1 MGLSPHLAVTLFCFLICTGNGIHGCN-DSPLREIINTLNQVTEKGTPCTEMFVPDVLTAT
Acomys 1 MGLRPQLATVLLCFLACTGDYIHGHN-DTALKEIIHTLNQVTEKGTPCTEMVVPDVLTAT


Homo 61 KNTTEKETFCRAATVLRQFYSHHEKDTRCLGATAQQFHRHKQLIRFLKRLDRNLWGLAGL
Mus 60 KNTTESELVCRASKVLRIFYLKHGK-TPCL-------KKNSSVLMELQRLFRAFRCLDSS
Rattus 60 RNTTENELICRASRVLRKFYFPRDV-PPCL-------KNKSGVLGELRKLCRGVSGLNSL
Acomys 60 KNSTEKELLCRASRVLRKFYFPHEV-TLCL-------KNNPKVLKDLKKLSRGISSLYPL


Homo 121 NSCPVKEANQSTLENFLERLKTIMREKYSKCS---S
Mus 112 ISCTMNESKSTSLKDFLESLKSIMQMDYS-------
Rattus 112 RSCTVNESTLTTLKDFLESLKSILRGKYLQSCTSMS
Acomys 112 ESCTVNESSYTTLKDFLERLRRIVQKKYCQC-----

**IL-5**

MAFFT Alignment

Homo 1 M-RMLLHLSLLALGAAYVYAIPTEIPTSALVKETLALLSTHRTLLIANETLRIPVPVHKN
Mus 1 MRRMLLHLSVLTLSC--VWATAMEIPMSTVVKETLTQLSAHRALLTSNETMRLPVPTHKN
Rattus 1 M-RMLLCLNVLTLSC--VWAIAMEIPMSTVVKETLIQLSTHRALLTSNETMRLPVPTHKN
Acomys 1 M-RVLLHLSFLTLPC--VWAVAMEIPMSAVVKETLIQLSAHRALLTSNETVRLPVPTHKN


Homo 60 HQLCTEEIFQGIGTLESQTVQGGTVERLFKNLSLIKKYIDGQKKKCGEERRRVNQFLDYL
Mus 59 HQLCIGEIFQGLDILKNQTVRGGTVEMLFQNLSLIKKYIDRQKEKCGEERRRTRQFLDYL
Rattus 58 HQLCIGEIFQGLDILKNQTVRGGTVEIPFQNLSLIKKYIDGQKEKCGEERRKTRHFLDYL
Acomys 58 HQLCIAEIFQGLDILKNQTARGGTVETLFQNLSLIKKYIDRQKEKCGEERRRTRQFLDYL


Homo 120 QEFLGVMNTEWIIE-
Mus 119 QEFLGVMSTEWAMEG
Rattus 118 QEFLGVMSTEWAMEV
Acomys 118 QEFLGVLSTEWTMDG

**IL-6**

MAFFT Alignment

Homo 1 MNSFSTSAFGPVAFSLGLLLVLPAAFP-APVPPGEDSKDVAAPHRQPLTSSERIDKQIRY
Mus 1 MKFLSARDFHPVAF-LGLMLVTTTAFPTSQVRRGDF-TEDTTPNRPVYTTS-QVGGLITH
Rattus 1 MKFLSARDFQPVAF-LGLMLLTATAFPTSQVRRGDF-TEDTTHNRPVYTTS-QVGGLITY
Acomys 1 MKFLSARDLHPLVF-LGLMLVTAAAFPTSQVRRGDLATADTTPNRPVYTTSQQVGGLVTN


Homo 60 ILDGISALRKETCNKSNMCESSKEALAENNLNLPKMAEKDGCFQSGFNEETCLVKIITGL
Mus 58 VLWEIVEMRKELCNGNSDCMNNDDALAENNLKLPEIQRNDGCYQTGYNQEICLLKISSGL
Rattus 58 VLREILEMRKELCNGNSDCMNSDDALSENNLKLPEIQRNDGCFQTGYNQEICLLKICSGL
Acomys 60 VLKEVLEMRKELCNGSSDCMNNEDALSENRLNFPVIQINDGCFETKHDWEICLLKITSGL


Homo 120 LEFEVYLEYLQNRFE-SSEEQARAVQMSTKVLIQFLQKKAKNLDAITTPDPTTNASLLTK
Mus 118 LEYHSYLEYMKNNLKDNKKDKARVLQRDTETLIHIFNQEVKDLHKIVLPTPISNALLTDK
Rattus 118 LEFRFYLEFVKNNLQDNKKDKARVIQSNTETLVHIFKQEIKDSYKIVLPTPTSNALLMEK
Acomys 120 LEYEIYLEYVKNNIQDSKKEKARVIQTSTQALINILRQEVKEPGKIISPGPTSTALQMET


Homo 179 LQAQNQWLQDMTTHLILRSFKEFLQSSLRALRQM
Mus 178 LESQKEWLRTKTIQFILKSLEEFLKVTLRSTRQT
Rattus 178 LESQKEWLRTKTIQLILKALEEFLKVTMRSTRQT
Acomys 180 LKPQNEWLRTKITQLILKALEEFLKDTMRSTRKS

**IL-10**

MAFFT Alignment

Homo 1 MHSSALLCCLVLLTGVRASPGQGTQSENSCTHFPGNLPNMLRDLRDAFSRVKTFFQMKDQ
Mus 1 MPGSALLCCLLLLTGMRISRGQYSREDNNCTHFPVGQSHMLLELRTAFSQVKTFFQTKDQ
Rattus 1 MPGSALLCCLLLLAGVKTSKGHSIRGDNNCTHFPVSQTHMLRELRAAFSQVKTFFQKKDQ
Acomys 1 MPGSALLCCLILLAGVGTSRG-----EYNCTHFAVSQTHMLRELRHAFSQVKTFFQKRDQ


Homo 61 LDNLLLKESLLEDFKGYLGCQALSEMIQFYLEEVMPQAENQDPDIKAHVNSLGENLKTLR
Mus 61 LDNILLTDSLMQDFKGYLGCQALSEMIQFYLVEVMPQAEKHGPEIKEHLNSLGEKLKTLR
Rattus 61 LDNILLTDSLLQDFKGYLGCQALSEMIKFYLVEVMPQAENHGPEIKEHLNSLGEKLKTLW
Acomys 56 LDNILLTDSLMQDFKGYLGCQALSEMIKFYLIEVMPQAENHGPEIKEHLNSLGEKLKTLR


Homo 121 LRLRRCHRFLPCENKSKAVEQVKNAFNKLQEKGIYKAMSEFDIFINYIEAYMTMKIRN
Mus 121 MRLRRCHRFLPCENKSKAVEQVKSDFNKLQDQGVYKAMNEFDIFINCIEAYMMIKMKS
Rattus 121 IQLRRCHRFLPCENKSKAVEQVKNDFNKLQDKGVYKAMNEFDIFINCIEAYVTLKMKN
Acomys 116 MRLQRCHRFFPCENKSKAVEQVKSDFNKLQEKGVYKAMSEFDIFINCIEAYLTIKMKN

**IL-12p35**

MAFFT Alignment

Homo 1 MWPPGSASQPPPSPAAATGLHPAARPVSLQCRLSMCPARSLLLVATLVLLDHLSLARNLP
Mus 1 M-----VSVPTASPSASSS--------SSQCRSSMCQSRYLLFLATLALLNHLSLARVIP
Rattus 1 ----------------------------------MCQSRYLLFLATLVLLNHLTSARVIP
Acomys 1 ----------------------------------MCQSRRPLFLA-IVVLTYLSLARANP


Homo 61 V-ATPDPGMFPCLHHSQNLLRAVSNMLQKARQTLEFYPCTSEEIDHEDITKDKTSTVEAC
Mus 48 V-SGP----ARCLSQSRNLLKTTDDMVKTAREKLKHYSCTAEDIDHEDITRDQTSTLKTC
Rattus 27 V-SGP----AKCLNQSQNLLKTTDDMVRTAREKLKHYSCTAGDIDHEDITRDKTSTLEAC
Acomys 26 VHHDP----AQCLHHAQNLLKATDNMMKTAREKLRHYSCTPGDIDHEDITRDKTSTLKAC


Homo 120 LPLELTKNESCLNSRETSFITNGSCLASRKTSFMMALCLSSIYEDLKMYQVEFKTMNAKL
Mus 103 LPLELHKNESCLATRETSSTTRGSCLPPQKTSLMMTLCLGSIYEDLKMYQTEFQAINAAL
Rattus 82 LPLELHKNESCLATKETSSIIRGSCLPPQKTSLMMTLCLGSIYEDLKMYQSEFQAINAAL
Acomys 82 LPLELAKNESCVATGETSSTIRGSCLPPQKASWMMTLCLSSIYEDLKMYQTEFQAIKAEL


Homo 180 LMDPKRQIFLDQNMLAVIDELMQALNFNSETVPQKSSLEEPDFYKTKIKLCILLHAFRIR
Mus 163 QNHNHQQIILDKGMLVAIDELMQSLNHNGETLRQKPPVGEADPYRVKMKLCILLHAFSTR
Rattus 142 QSHNHQQITLDRNMLMAIDELMRSLNHSGETLHQKAPMGEADPYRVKMKLCILLHAFSTR
Acomys 142 QSHNQQQITLREDMLAAIKELMRTLNPNGETPSQTPAPERADPYKVKIKLCILLHAFSIR


Homo 240 AVTIDRVMSYLNAS
Mus 223 VVTINRVMGYLSSA
Rattus 202 VMTINRVMNYLSSS
Acomys 202 VTTINKVMSYLNSS

**IL-12p40**

MAFFT Alignment

Homo 1 MCHQQLVISWFSLVFLASPLVAIWELKKDVYVVELDWYPDAPGEMVVLTCDTPEEDGITW
Mus 1 MCPQKLTISWFAIVLLVSPLMAMWELEKDVYVVEVDWTPDAPGETVNLTCDTPEEDDITW
Rattus 1 MCHQKLTFSWFAMVLLVSPLMAMWELEKDVYVVEVDWRPDAPGETVTLTCDSPEEDDITW
Acomys 1 MCHQKLTISWFAVVLLASPLMAIWELEKDVYVVEVDWSPGAPGESVALTCDTPEEDDITW


Homo 61 TLDQSSEVLGSGKTLTIQVKEFGDAGQYTCHKGGEVLSHSLLLLHKKEDGIWSTDILKDQ
Mus 61 TSDQRHGVIGSGKTLTITVKEFLDAGQYTCHKGGETLSHSHLLLHKKENGIWSTEILKN-
Rattus 61 TSDQRRGVIGSGKTLTITVREFLDAGQYTCHRGGETLSHSHLLLHKKENGIWSTEILKN-
Acomys 61 TSDQRIDFKESGKTVTFEVKEFIHAGQYTCHKGDETLSHSRLLLHKKENGIWSTDILKD-


Homo 121 KEPKNKTFLRCEAKNYSGRFTCWWLTTISTDLTFSVKSSRGSSDPQGVTCGAATLSAERV
Mus 120 --FKNKTFLKCEAPNYSGRFTCSWLVQRNMDLKFNIKSSSSSPDSRAVTCGMASLSAEKV
Rattus 120 --FKNKTFLKCEAPNYSGRFTCSWLVHRNTDLKFNIKSSSSSPESRAVTCGRASLSAEKV
Acomys 120 --SKNKTFLRCEAPNYSGRFTCSWLAER-TDLKFSIKSGSSSPDSRAVTCGAASLSSEKV


Homo 181 RGDNKEYE-YSVECQEDSACPAAEESLPIEVMVDAVHKLKYENYTSSFFIRDIIKPDPPK
Mus 178 TLDQRDYEKYSVSCQEDVTCPTAEETLPIELALEARQQNKYENYSTSFFIRDIIKPDPPK
Rattus 178 TLNQRDYEKYSVACQEDVTCPTAEETLPIELVVEAQQQNKYENYSTSFFIRDIIKPDPPK
Acomys 177 RVDEQDFEQYSVSCQEDVACPTAEETLPIELVLDAQEQNKYENYSTSFFIRDIIKPDPPK


Homo 240 NLQLKPLKNSRQVEVSWEYPDTWSTPHSYFSLTFCVQVQGKSKREK--------------
Mus 238 NLQMKPLKNS-QVEVSWEYPDSWSTPHSYFSLKFFVRIQRKKEKMK--------ETEEGC
Rattus 238 NLQVKPLKNS-QVEVSWEYPDSWSTPHSYFSLKFFVRIQRKKEKTK--------ETEEEC
Acomys 237 NVQVKPLKND-EVEVSWEYPDSWSTPHSYFSLKFFVQIQCNKERTKKKRKKTEVEIERKC


Homo 286 --KDRVFTDKTSATVICRKNASISVRAQDRYYSSSWSEWASVPCS---------------
Mus 289 NQKGAFLVEKTSTEVQC-KGGNVCVQAQDRYYNSSCSKWACVPCRVRS------------
Rattus 289 NQKGAFLVEKTSAEVQC-KGANICVQAQDRYYNSSCSKWTCVPCRGRS------------
Acomys 296 KQKEPFLVDKTSATVEC-KGAKVCVQAQDRYYNSSFSKWECVPCKIQSQDATLEGKEVED


Homo -----------------------------
Mus -----------------------------
Rattus -----------------------------
Acomys 355 NRKEKFHLMMEETPKAIFCLAAFFQFLGS

**IL-17**

MAFFT Alignment

Homo 1 MTPGKTSLVS--LLLLLSLEAIVKAGITIPRNPGCPNSEDKNFPRTVMVNLNIHNR-NTN
Mus 1 MSPGRASSVSLMLLLLLSLAATVKAAAIIPQSSACPNTEAKDFLQNVKVNLKVFNSLGAK
Rattus 1 MSPRRIPSMCLMLLLLLNLEATVKAAVLIPQSSVCPNAEANNFLQNVKVNLKVLNSLSSK
Acomys 1 MSLGKTSSVSLLLLLLLSLEAAVNAGTLVPQGTVCPNTDTKSFLQDVKVNLKILNSFSPR


Homo 58 TNPKRSSDYYNRSTSPWNLHRNEDPERYPSVIWEAKCRHLGCINADGNVDYHMNSVPIQQ
Mus 61 VSSRRPSDYLNRSTSPWTLHRNEDPDRYPSVIWEAQCRHQRCVNAEGKLDHHMNSVLIQQ
Rattus 61 ASSRRPSDYLNRSTSPWTLSRNEDPDRYPSVIWEAQCRHQRCVNAEGKLDHHMNSVLIQQ
Acomys 61 VNSRRPSDYLNRSTSPWTLHRNEDPDRYPPVIWEARCRHQRCVNAEGKLDHHMNSVLIQQ


Homo 118 EILVLRREPPHCPNSFRLEKILVSVGCTCVTPIVHHVA
Mus 121 EILVLKREPESCPFTFRVEKMLVGVGCTCVASIVRQAA
Rattus 121 EILVLKREPEKCPFTFRVEKMLVGVGCTCVSSIVRHAS
Acomys 121 EILVLRREAEKCPLSFRLEKMLVGVGCTCVSSIVRHVA

**CCL2**

MAFFT Alignment

Homo 1 MKVSAVLLCLLLMTAAFNPQGLAQPDALNVPSTCCFTFSSKKISLQRLKSYV-ITTSRCP
Mus 1 MQVPVMLLGLLFTVAGWSIHVLAQPDAVNAPLTCCYSFTSKMIPMSRLESYKRITSSRCP
Rattus 1 MQVSVTLLGLLFTVAACSIHVLSQPDAVNAPLTCCYSFTGKMIPMSRLENYKRITSSRCP
Acomys 1 MQVSAKLLCLLLTAAASSSPMLAQPDSVTSPRTCCYSFTSKRIPLQRLESYKRITSSKCP


Homo 60 QKAVIFRTKLGKEICADPKEKWVQNYMKHLGRK------------AHTLKT---------
Mus 61 KEAVVFVTKLKREVCADPKKEWVQTYIKNLDRNQMRSEPTTLFKTASALRSSAPLN-VKL
Rattus 61 KEAVVFVTKLKREICADPNKEWVQKYIRKLDQNQVRSETTVFYKIASTLRTSAPLN-VNL
Acomys 61 KEAIIFVTKLKKEICADPTMDWVQSYIQKLDQNQRKSEATAVFKTASSPGSSASLNAANS


Homo ------------------------------
Mus 120 TRKSEANAST-TFSTTTSSTSVGVTSVTVN
Rattus 120 THKSEANAST-LFSTTTSSTSVEVTSMTEN
Acomys 121 THKPSANASTATFPTATSSTSVGVTSVTVN

**CCL3**

MAFFT Alignment

Homo 1 MQVSTAALAVLLCTMALCNQVLSAPLAADTPTACCFSYTSRQIPQNFIADYFETSSQCSK
Mus 1 MKVSTTALAVLLCTMTLCNQVFSAPYGADTPTACCFSY-SRKIPRQFIVDYFETSSLCSQ
Rattus 1 MKVSTAALAVLLCTMALWNEVFSAPYGADTPTACCFSY-GRQIPRKFIADYFETSSLCSQ
Acomys 1 MKVPTAALAVLLCTMALCNQVFSAPYGADTPTACCFSY-SRQIPRKFIVDYFETSSLCSQ


Homo 61 PSVIFLTKRGRQVCADPSEEWVQKYVSDLELSA
Mus 60 PGVIFLTKRNRQICADSKETWVQEYITDLELNA
Rattus 60 PGVIFLTKRNRQICADPKETWVQEYITELELNA
Acomys 60 PGVIFLTKRNRQICADPKETWVQEYITDLELNA

**CCL5**

MAFFT Alignment

Homo 1 MKVS-AAALAVILIATALCAPASASPYSSDTTPCCFAYIARPLPRAHIKEYFYTSGKCSN
Mus 1 MKIS-AAALTIILTAAALCTPAPASPYGSDTTPCCFAYLSLALPRAHVKEYFYTSSKCSN
Rattus 1 MKISTAASLTVILVAAALCTPAPASPYGSDTTPCCFAYLSLALPRAHVKEYFYTSSKCSN
Acomys 1 MKIS-AAALAVIVTAAAICAPASASPYGSDTAPCCFAYHSRVLPRDHVKEYFYTSSKCSN


Homo 60 PAVVFVTRKNRQVCANPEKKWVREYINSLEMS
Mus 60 LAVVFVTRRNRQVCANPEKKWVQEYINYLEMS
Rattus 61 LAVVFVTRRNRQVCANPEKKWVQEYINYLEMS
Acomys 60 LAVIFVTRRNRQVCANPEKKWVRKYINYLEMK

**CSF2**

MAFFT Alignment

Homo 1 MWLQSLLLLGTVACSISAPARSPSPSTQPWEHVNAIQEARRLLNLSRDTAA---EMNETV
Mus 1 MWLQNLLFLGIVVYSLSAPTRSPITVTRPWKHVEAIKEALNLLD---DMPV---TLNEEV
Rattus 1 MWLQNLLFLGIVVYSFSAPTRSPNPVTRPWKHVDAIKEALSLLN---DMRALENEKNEDV
Acomys 1 MWLQSLLFLSIVVCSFSAPTRSPVPVTRPWKHVDAIIEALSLLN---EMPV---TVTENV


Homo 58 EVISEMFDLQEPTCLQTRLELYKQGLRGSLTKLKGPLTMMASHYKQHCPPTPETSCATQI
Mus 55 EVVSNEFSFKKLTCVQTRLKIFEQGLRGNFTKLKGALNMTASYYQTYCPPTPETDCETQV
Rattus 58 DIISNEFSIQRPTCVQTRLKLYKQGLRGNLTKLNGALTMIASHYQTNCPPTPETDCEIEV
Acomys 55 GVVSNDFSIQNPTCVQTRLDLYKQGLRGNFTRLEGDLTVIASHYKKNCPPTPETNCESQV


Homo 118 ITFESFKENLKDFLLVIPFDCWEPVQE
Mus 115 TTYADFIDSLKTFLTDIPFECKKPGQK
Rattus 118 TTFEDFIKNLKGFLFDIPFDCWKPVQK
Acomys 115 IIYEEFIENLKGFLMVIPFDCWKSAQK

**IFNγ**

MAFFT Alignment

Homo 1 MKYTSYILAFQLCIVLGSLGCYCQDPYVKEAENLKKYFNAGHSDVADNGTLFLGILKNWK
Mus 1 MNATHCILALQLFLMAVS-GCYCHGTVIESLESLNNYFNSSGID-VEEKSLFLDIWRNWQ
Rattus 1 MSATRRVLVLQLCLMALS-GCYCQGTLIESLESLKNYFNSSSMDAMEGKSLLLDIWRNWQ
Acomys 1 MNAKHCILALQFCLMAIY-GCYCQGTVIEETTNLKEYF-ASSISVSNGEDLLLHILRNWQ


Homo 61 EESDRKIMQSQIVSFYFKLFKNFKDDQSIQKSVETIKEDMNVKFFNSNKKKRDDFEKLTN
Mus 59 KDGDMKILQSQIISFYLRLFEVLKDNQAISNNISVIESHLITTFFSNSKAKKDAFMSIAK
Rattus 60 KDGNTKILESQIISFYLRLFEVLKDNQAISNNISVIESHLITNFFSNSKAKKDAFMSIAK
Acomys 59 QDGDTKTIDIQIISFYFKLFEALKGHKAIQRSIDTIRADLIANFFNNSEEKYNGFMRIAK


Homo 121 YSVTDLNVQRKAIHELIQVMAELSPAAKTGKRKRSQMLF--------RGRRASQ
Mus 119 FEVNNPQVQRQAFNELIRVVHQLLPESSLRKRKRSRC-----------------
Rattus 120 FEVNNPQIQHKAVNELIRVIHQLSPESSLRKRKRSRC-----------------
Acomys 119 IEVNDPQNQRKAINELVTVMSHLSPKSKQRKRKRSRCCFGAGDRLNKNNPASTI

**TNFα**

MAFFT Alignment

Homo 1 MSTESMIRDVELAEEALPKKTGGPQGSRRCLFLSLFSFLIVAGATTLFCLLHFGVIGPQR
Mus 1 MSTESMIRDVELAEEALPQKMGGFQNSRRCLCLSLFSFLLVAGATTLFCLLNFGVIGPQR
Rattus 1 MSTESMIRDVELAEEALPKKMGGLQNSRRCLCLSLFSFLLVAGATTLFCLLNFGVIGPNK
Acomys 1 MSTESMIRDVELAEEALPQKAGSPQNSRRCLCLSLFSFLLVAGATALFCLLNFGVIGPQR


Homo 61 EE-FPRDLSLISPLAQ--AVRSSSRTPSDKPVAHVVANPQAEGQLQWLNRRANALLANGV
Mus 61 DEKFPNGLPLISSMAQTLTLRSSSQNSSDKPVAHVVANHQVEEQLEWLSQRANALLANGM
Rattus 61 EEKFPNGLPLISSMAQTLTLRSSSQNSSDKPVAHVVANHQAEEQLEWLSQRANALLANGM
Acomys 61 EEKFPNGLPIIGSMAQTLTLRSSSQNSSDKPVAHVVANHQVEEQLEWLSRRANALLANGM


Homo 118 ELRDNQLVVPSEGLYLIYSQVLFKGQGCPSTHVLLTHTISRIAVSYQTKVNLLSAIKSPC
Mus 121 DLKDNQLVVPADGLYLVYSQVLFKGQGCPD-YVLLTHTVSRFAISYQEKVNLLSAVKSPC
Rattus 121 DLKDNQLVVPADGLYLIYSQVLFKGQGCPD-YVLLTHTVSRFAISYQEKVSLLSAIKSPC
Acomys 121 DLKDNQLVVPSDGLYLVYSQVLFKGQGCPN-YVLLTHTVSRFAVSYEDKVNLLSAIKSPC


Homo 178 QRETPEGAEAKPWYEPIYLGGVFQLEKGDRLSAEINRPDYLDFAESGQVYFGIIAL
Mus 180 PKDTPEGAELKPWYEPIYLGGVFQLEKGDQLSAEVNLPKYLDFAESGQVYFGVIAL
Rattus 180 PKDTPEGAELKPWYEPMYLGGVFQLEKGDLLSAEVNLPKYLDITESGQVYFGVIAL
Acomys 180 PKDTPEGAELKPWYEPIYLGGVFQLEKGDRLSAEVNLPKYLDFAESGQVYFGVIAL

**CXCL1**

MAFFT Alignment

Homo 1 MARATLSAAPSNPRLLRVALLLLLLVAASRRAAGAPLATELRCQCLQTLQGIHLKNIQSV
Mus 1 M-------IPATRSLLCAA---LLLLATSRLATGAPIANELRCQCLQTMAGIHLKNIQSL
Rattus 1 M-------VSATRSLLCAA---LPVLATSRQATGAPVANELRCQCLQTVAGIHFKNIQSL
Acomys 1 M-------APATSPLLRGTLLLLLLLATSPQATGAPVASELRCQCLQTVAGIHLKNIQSL


Homo 61 KVKSPGPHCAQTEVIATLKNGQKACLNPASPMVKKIIEKMLKNGKSN
Mus 51 KVLPSGPHCTQTEVIATLKNGREACLDPEAPLVQKIVQKMLK-GVPK
Rattus 51 KVMPPGPHCTQTEVIATLKNGREACLDPEAPMVQKIVQKMLK-GVPK
Acomys 54 KVTPPGPHCIQTEVIATLKNGREACLDPEAPMVRKVVQKMLN-GISK
